# Comparison of the efficacy and safety of 10 glucagon-like peptide-1 receptor agonists as add-on to metformin in patients with type 2 diabetes: a systematic review

**DOI:** 10.3389/fendo.2023.1244432

**Published:** 2023-08-28

**Authors:** Zeyu Xie, Jia Hu, Hangye Gu, Mengting Li, Jisheng Chen

**Affiliations:** Key Specialty of Clinical Pharmacy, The First Affiliated Hospital of Guangdong Pharmaceutical University, Guangzhou, China

**Keywords:** glucagon-like peptide-1 receptor agonists, safety, efficacy, systematic review, randomized controlled trials, type 2 diabetes

## Abstract

**Purpose:**

This study aimed to perform a network meta-analysis to objectively evaluate the efficacy and safety of 10 Glucagon-like peptide-1 receptor agonists (GLP-1RAs) in combination with metformin that is approved for use worldwide in patients with type 2 diabetes and to provide evidence-based support and reference for the selection of clinical treatment.

**Methods:**

Three databases (PubMed, Embase, and Cochrane Library) were searched from their respective inception until September 30, 2022. Only randomized controlled trials comparing the efficacy and safety of GLP-1RAs for treating type 2 diabetes (T2D) were included. The 10 GLP-1RAs are exenatide (including exenatide twice daily and once weekly), liraglutide, lixisenatide, dulaglutide, PEX168, semaglutide (subcutaneous and oral semaglutide), tirzepatide and albiglutide.

**Results:**

34 RCTs with 10 GLP-1RAs and 12993 patients were included in the Network Meta-Analysis (NMA). According to the NMA, tirzepatide 15 mg, semaglutide 1.0 mg, PEX168-200μg, oral semaglutide 14 and dulaglutide 1.5 mg reduced HbA1c by -2.23%, -1.57%, -1.12%, -1.10%, -1.09% and body weight by -11.33 kg, -5.99 kg, +0.40 kg, -3.95 kg, -1.87 kg, respectively. There was no significant difference in the rate of adverse events for tirzepatide 15 mg, oral-semaglutide 14 mg, and semaglutide 1.0 mg. PEX168-200μg, tirzepatide 15mg, and oral semaglutide 14mg had Surface Under the Cumulative Ranking (SUCRA) values greater than placebo, and only tirzepatide 15mg and oral semaglutide 14mg were significantly different from placebo in the rate of serious adverse events. All GLP-1RA did not lead to increased incidence of hypoglycemia. Albiglutide 30mg and semaglutide 1.0mg significantly differed from placebo in Adverse Event (AE) withdrawal. Finally, the sensitivity analysis and publication bias analysis results indicate that the study results are reliable.

**Conclusion:**

This study’s results showed that GLP-1RAs were effective in lowering HbA1c and reducing body weight without increased incidence of hypoglycemic reactions. In addition, this study may provide reference and evidence-based medical evidence for clinicians to select GLP-1RAs in patients with T2D and high body mass index (BMI). Based on the NMA results, tirzepatide 15mg and semaglutide 1.0mg may be preferred.

## 1 Introduction

As people’s diets and lifestyles change, diabetes prevalence is increasing, with type 2 diabetes accounting for 90-95% of all cases of diabetes. Diabetes imposes a heavy economic burden on individuals and society, with the annual cost of diagnosing diabetes estimated at $327 billion in 2017 in the US (1). Diabetes prevalence is significantly higher in people who are overweight or obese. Studies have shown that the higher the BMI, the higher the risk of type 2 diabetes (2–4), and patients with pre-diabetes often have associated cardiovascular risk factors, including hypertension and dyslipidemia, and may be at increased risk of cardiovascular disease (5–7).

The American Diabetes Association (ADA) Medical Standards of Care for Diabetes 2022 Edition recommends metformin as the first-line treatment and GLP-1RAs as part of the treatment regimen for patients with T2D in combination with atherosclerotic cardiovascular disease or high-risk factors, renal disease, or heart failure (8). GLP-1RA has been shown to improve hemoglobin A1c (HbA1c), reduce body weight, and have cardiovascular benefits, and should be more widely used in clinical practice (9–15).

In 2019, 48% of diabetes deaths occurred before age 70. Elevated blood glucose contributes to about 20% of deaths from cardiovascular disease (16). In 2021, approximately 537 million adults (aged 20-79) worldwide had diabetes, and an estimated 6.7 million adults will have died from diabetes or its complications (17). However, in the CAPTURE study (18) (a multinational, cross-sectional study of cardiovascular disease prevalence in adults with type 2 diabetes across 13 countries), results showed that the prevalence of cardiovascular disease in Chinese patients with T2DM was 33.9%, of which 94.9% was atherosclerotic cardiovascular disease (ASCVD). Only 1.5% of Chinese patients with T2DM and combined ASCVD were treated with GLP-1RAs with cardiovascular benefit, a large gap between clinical practice and guideline recommendations (19, 20).

An NMA of metformin in combination of GLP-1RAs has not been previously performed. Therefore, we sought to objectively evaluate the efficacy and safety of 10 globally approved and marketed GLP-1RAs in combination with metformin for treating patients with T2DM through NMA and to provide evidence-based support and reference for the clinical selection of GLP-1RAs.

## 2 Methods

### 2.1 Registration

The Preferred Items report for this systematic review, the NMA for Systematic Reviews and Meta-Analyses (PRISMA) statement, and the PRISMA checklist is provided in **Supplementary File 1**. The protocol was registered in the International Prospective Register of Systematic Reviews (PROSPERO) (registration number: CRD42023390347).

### 2.2 Data source

The Cochrane Library, PubMed, and Embase databases were searched from the creation of the databases to September 30, 2022, with no language restrictions. We used search terms including “diabetes mellitus, type 2”, “type 2 diabetes”, “glucagon-like peptide-1 receptor agonist”, “GLP-1 receptor agonist”, and “randomized controlled trial,” including any of these terms in any field. The search strategy, including all search terms, is shown in **Supplementary File 2**.

### 2.3 Inclusion criteria

Inclusion and exclusion criteria were developed according to the principles of Participants, Intervention, Comparison, Outcomes, and Study (PICOS). Inclusion criteria: (1) Participants: Patients diagnosed with type 2 diabetes, HbA1c ≥7.0%, age ≥18 years, no diabetes-related complications, no gender or race restrictions, and drug treatment cycle ≥12 weeks (initial dose + maintenance dose). (2) Intervention: 10 GLP-1RAs (“exenatide (including exenatide twice daily and once weekly)” or “liraglutide” or “lixisenatide” or “dulaglutide” or “loxenatide” or “PEX168” or “semaglutide (including oral semaglutide)” or “tirzepatide” or “albiglutide”) +/- other oral antidiabetic drugs (OADs) were taken in the treatment group. (3) Comparison: Placebo or another GLP-1RA in the control group. (4) Outcomes: Primary outcomes were HbA1c and rate of all adverse events. Secondary outcomes were weight loss, serious adverse events, hypoglycemic events, and withdrawal due to adverse events. (5) Study: The study type was RCT.

### 2.4 Exclusion criteria

Exclusion criteria: (1) The article was republished, case report, review, conference, monotherapy, animal studies, retrospective studies, and data could not be extracted. (2) The patient’s age <18, HbA1c<7.0%, and treatment cycle <12 weeks, presence of associated diabetic complications. (3) High-risk trials and type of non-randomized controlled trials.

### 2.5 Literature screening and data extraction

The Note Express software has been used to eliminate duplicates of articles, then read the titles and abstracts to exclude articles that did not meet the inclusion criteria, and finally read the full text according to the predefined inclusion and exclusion criteria to determine the final included studies, and extracted data from the included studies. Two investigators extracted data independently (Gu and Hu), and a third investigator resolved conflicting data (Chen). The extracted data include the following information: (1) study characteristics (e.g., year of publication, first author, mean age, sex, mean HbA1c (%), and total number of people included in the study). (2) Therapeutic interventions (e.g., drugs, dose, and cycle). (3) Clinical data (e.g., HbA1c reduction (%), weight loss (kg), number of adverse events, serious adverse events, withdrawals due to adverse events, and hypoglycemia events).

### 2.6 Risk of bias assessment

The risk of bias in the included studies was assessed independently by two investigators (Gu and Hu) using the software Review Manager 5.4.1 according to the Cochrane Risk of Bias Assessment Tool criteria, and a third investigator (Chen) adjudicated conflicting studies.

### 2.7 Analysis of data

Frequentist NMA was performed using software (Stata 16.1). The mean difference (MD) value was used to calculate continuous variables. Each effect size was expressed as a 95% confidence interval (95%_CI). The odds ratio (OR) value is used to calculate dichotomous variables (the number of adverse events), and a higher value means more adverse events, which means worse outcomes. We used the surface under the cumulative ranking (SUCRA) to rank the outcome of each treatment and finally expressed it as a percentage. For heterogeneity results of each outcome, we used software (Review Manager 5.4.1) to calculate, and the results are expressed in I^2^ and p-value (if p-value for Q test < 0.10 or I^2^ > 50% was defined as significant heterogeneity).

For the inconsistency test of the whole network meta, we used software (Stata 16.1) to test global inconsistency, local inconsistency (node splitting method), and closed-loop inconsistency (If the p-value is greater than 0.05, it means that the inconsistency is not significantly different). If the results are consistent (p>0.05), the results of the network meta are reliable. Meanwhile, we evaluated the included studies by drawing the network plots, funnel plots of outcome indicators, and risk of publication bias plots. In addition, a cluster analysis was performed to compare the effect of GLP-1RAs on efficacy (reduction in HbA1c) and safety (rate of adverse events). Finally, a sensitivity analysis is required if the included studies are high-risk.

## 3 Result

### 3.1 Inclusion process and study characteristics

**Figure 1:**
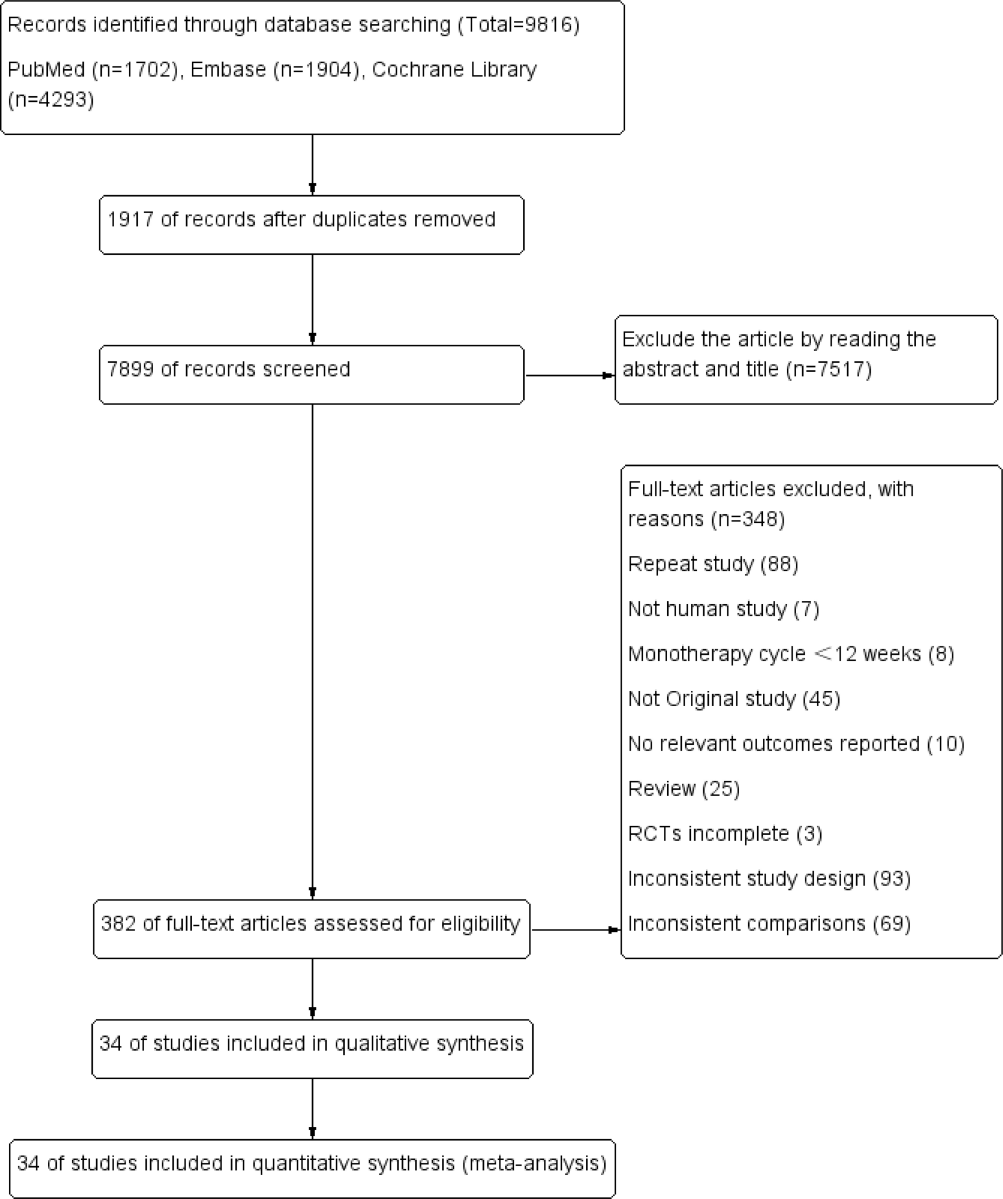
Flow chart of the study selection process.

The study’s screening process and the patients’ characteristics included are shown in **Figure 1**; **Table 1**. A total of 9816 articles were initially included by searching the databases according to the predefined search criteria, including PubMed (1702), Embase (1904), and the Cochrane Library (4293), further removing duplicates (7517) and reading the full text and abstracts (348). Finally, 34 studies with 11 treatments (involving 12993 patients) were included in the NMA. In the 34 included studies, 18 used metformin + GLP-1RA as the therapeutic agent, and 16 used metformin + GLP-1RA ± OAD (e.g., sulfonylurea, thiazolidinedione, and sodium-glucose cotransporter-2 inhibitors).

**Table 1:** Patient basic characteristics of included studies.

Study ID	Year± SD	N_Total_	N_Male_	Baseline (HbA1c (%))	Weeks	Combination Therapy Drugs	Intervention	HbA1c (%)		Weight (kg)		Safety				
								N_1_	Mean ±SD	N_2_	Mean ±SD	N_3_	N_T_	N_S_	N_H_	N_AE_
01 Bo Ahrén 2014 (20)	54.3 (10.1)	403	185	8.1 (0.8)	104	Metformin	Albi 30mg	297	-0.63 (0.07)	296	-1.21 (0.24)	302	253	36	13	20
							Placebo	100	0.27 (0.11)	100	-1.00 (0.41)	101	80	13	5	5
02 P D Home 2015 (21)	54.5 (9.5)	386	205	8.2 (0.9)	52	Metformin+ SU	Albi 30mg	269	-0.55 (0.06)	269	-0.42 (0.24)	271	216	17	57	12
							Placebo	115	0.33 (0.08)	115	-0.40 (0.36)	115	80	7	13	6
03 Fei Gao 2020 (22)	52.8 (10.6)	354	204	8.5 (0.9)	24	Metformin	PEX168 200μg	175	-1.14 (0.08)	175	-0.40 (0.34)	178	85	5	1	2
							Placebo	179	-0.35% (0.08)	179	-0.80 (0.41)	182	78	3	1	3
04 Xiaoping Chen 2017 (23)	49.8 (10.9)	78	48	8.3 (1.2)	12	Metformin	PEX168 200μg	40	-1.36 (0.32)	NA		40	18	NA	0	NA
							Placebo	38	0.13 (0.32)	NA		38	10	NA	0	NA
05 Michael Nauck 2016 (24)	56.2 (10.3)	404	244	8.4 (0.7)	26	Metformin	Lixisenatide 20μg	191	-1.238 (1.01)	191	-3.69 (4.75)	202	129	7	5	15
							Liraglutide 1.8mg	194	-1.809 (0.92)	194	-4.24 (4.27)	202	145	12	3	13
06 Chang Yu Pan 2014 (25)	54.5 (10.3)	390	200	7.9 (0.8)	24	Metformin± SU	Lixisenatide 20μg	196	-0.83 (0.10)	196	-1.50 (0.27)	196	126	3	11	11
							Placebo	194	-0.47 (0.10)	194	-1.24 (0.27)	194	92	4	5	3
07 G B Bolli 2014 (26)	56.1 (9.3)	482	215	8.0 (0.8)	24	Metformin	Lixisenatide 20μg	308	-0.88 (0.10)	313	-2.65 (0.39)	322	223	12	7	22
							Placebo	158	-0.42 (0.10)	158	-1.63 (0.39)	160	105	4	1	4
08 Julio Rosenstock 2013 (27)	57.4 (9.9)	634	338	8.0 (0.8)	24	Metformin	Lixisenatide 20μg	295	-0.79 (0.05)	295	-2.96 (0.23)	318	221	9	8	33
							Exenatide 10μg	297	-0.96 (0.05)	296	-3.98 (0.23)	316	228	7	25	41
09 Bo Ahrén 2013 (28)	54.5 (9.2)	680	293	8.0 (0.9)	24	Metformin	Lixisenatide 20μg	483	-0.81 (0.07)	497	-2.02 (0.24)	510	354	13	19	32
							Placebo	164	-0.38 (0.08)	168	-1.64 (0.27)	170	102	2	1	2
10 Juan P Frías 2021 (29)	56.6 (10.4)	939	500	8.3 (1.0)	40	Metformin	Tirzepatide 15mg	464	-2.46 (0.05)	464	-12.40 (0.34)	470	324	27	8	40
							Semaglutide 1.0mg	461	-1.86 (0.05)	462	-6.20 (0.33)	469	301	13	2	19
11 Juan Pablo Frias 2018 (30)	58.7 (7.8)	158	75	8.1 (1.0)	26	Metformin	Tirzepatide 15mg	35	-2.4 (0.17)	35	-11.30 (0.88)	53	45	2	4	13
							Dulaglutide 1.5mg	47	-1.1 (0.15)	47	-2.70 (0.78)	54	40	3	2	6
							Placebo	41	0.1 (0.16)	41	-0.40 (0.81)	51	27	2	2	2
12 Tim Heise MD 2022 (31)	61.1 (7.1)	117	89	7.83 (0.72)	28	Metformin ±OAD	Tirzepatide 15mg	45	-2.06 (0.11)	45	-11.20 (0.90)	45	43	1	3	1
							Semaglutide 1.0mg	44	-1.52 (0.10)	44	-6.90 (0.90)	44	43	0	1	0
							Placebo	28	+0.19 (0.19)	28	0 (1.10)	28	22	2	0	3
13 Juan Pablo Frias 2020 (32)	56.6 (9.2)	82	51	8.4 (1.1)	12	Metformin	Tirzepatide 15mg	49	-1.9 (0.19)	49	-5.60 (0.79)	56	43	0	10	1
							Placebo	26	0.2 (0.21)	20	-0.50 (0.86)	26	13	0	0	1
14 Juan P Frias 2019 (33)	57.7 (9.8)	162	87	8.0 (0.8)	18	Metformin	Dulaglutide 1.5 mg	81	-1.23 (0.10)	81	-2.80 (0.39)	81	54	3	2	5
							Placebo	82	-0.44 (0.10)	82	-1.60 (0.39)	82	47	4	0	4
15 R S Weinstock 2015 (34)	53.66 (10.0)	481	236	8.1 (1.1)	26	Metformin	Dulaglutide 1.5 mg	301	-1.22 (0.05)	303	-3.18 (0.18)	NA				
							Placebo	176	0.03 (0.07)	177	-1.47 (0.24)	NA				
16 Kathleen M Dungan 2014 (35)	56.5 (9.3)	599	287	8.1 (0.8)	26	Metformin	Dulaglutide 1.5mg	279	-1.42 (0.05)	299	-2.90 (0.22)	299	185	5	26	18
							Liraglutide 1.8mg	272	-1.36 (0.05)	299	-3.61 (0.22)	300	189	11	17	18
17 Carol Wysham 2014 (36)	56 (10.0)	696	402	8.1 (1.3)	26	Metformin ±SLGT2	dulaglutide 1.5 mg	279	-1.51 (0.06)	279	-1.30 (0.29)	279	215	12	29	8
							Exenatide 10μg	276	-0.99 (0.06)	276	-1.07 (0.29)	276	198	15	44	9
							Placebo	141	-0.46 (0.08)	141	1.24 (0.37)	141	104	12	5	3
18 Richard Pratley 2019 (37)	56 (10.0)	711	370	8.0 (0.7)	26	Metformin ±SLGT2	Oral Semaglutide 14 mg	278	-1.2 (0.90)	278	-4.40 (4.40)	285	229	31	2	22
							Liraglutide 1.8 mg	272	-1.1 (0.90)	271	-3.20 (3.70)	284	211	22	7	17
							Placebo	134	-0.1 (0.70)	134	-0.60 (3.10)	142	95	15	3	3
19 Richard E Pratley 2018 (38)	55 (10.6)	699	333	8.2 (0.9)	40	Metformin	Semaglutide 1.0 mg	300	-1.78 (0.06)	300	-6.53 (0.28)	300	221	22	5	20
							Dulaglutide 1.5 mg	299	-1.37 (0.06)	299	-2.98 (0.27)	299	207	23	5	29
20 Andrew J Ahmann 2018 (39)	56.6 (24.4)	809	447	8.3 (1.1)	56	Metformin ±TZD/SU	Semaglutide 1.0 mg	404	-1.54 (0.06)	404	-5.63 (0.29)	404	303	38	33	38
							Exenatide 2.0 mg	405	-0.92 (0.06)	405	-1.85 (0.29)	405	309	24	33	29
21 Huub J van Eyk 2019 (40)	55 (11.0)	47	19	8.1 (0.9)	26	Metformin ±SU	Liraglutide 1.8 mg	22	−0.8 (1.00)	22	− 3.90 (3.60)	NA				
							Placebo	25	−0.6 (0.80)	25	− 0.60 (2.20)	NA				
22 Michael Nauck 2009 (41)	56.8 (9.4)	363	214	8.4 (0.9)	26	Metformin	Liraglutide 1.8 mg	236	-1.00 (0.07)	241	-2.79 (0.23)	242	158	16	9	31
							Placebo	120	0.09 (0.09)	121	-1.51 (0.31)	122	44	9	6	16
23 Bernard Zinman 2009 (42)	55 (11.0)	200	113	8.6 (1.2)	26	Metformin ±TZD	Liraglutide 1.8 mg	100	-1.56 (0.10)	100	-2.00 (0.30)	100	NA	7	8	17
							Placebo	100	-0.5 (0.10)	100	0.60 (0.30)	100	NA	12	5	6
24 D Russell-Jones 2009 (43)	57.6 (9.5)	244	187	8.3 (0.9)	26	Metformin ±SU	Liraglutide 1.8 mg	230	-1.33 (0.09)	230	-1.8 (0.33)	230	151	9	63	11
							Placebo	114	-0.24 (0.11)	114	-0.42 (0.39)	114	64	8	19	1
25 John B Buse 2009 (44)	56.3 (9.8)	464	241	8.2 (1.0)	26	Metformin ±SU	Liraglutide 1.8 mg	227	-1.12 (0.08)	231	-3.24 (0.33)	233	174	12	60	23
							Exenatide-10μg	226	-0.79 (0.08)	229	-2.87 (0.33)	231	182	6	78	31
26 Chieh-Hsiang Lu 2013 (45)	50.5 (9.0)	50	27	8.1 (1.0)	16	Metformin ±SU	Exenatide 10μg	26	-0.8 (0.60)	26	-2.00 (1.10)	26	13	NA	12	1
							Placebo	24	-0.1 (0.60)	24	-0.40 (1.00)	24	9	NA	1	0
27 G Derosa 2012 (46)	57.0 (7.5)	171	88	8.0 (0.7)	52	Metformin	Exenatide 10μg	81	-1.2 (0.20)	81	-6.40 (1.20)	NA				
							Placebo	82	-0.4 (0.10)	82	-2.30 (1.10)	NA				
28 Caroline M Apovian 2010 (47)	54.5 (10.0)	194	73	7.7 (0.9)	24	Metformin ±SU	Exenatide 10μg	96	-1.2 (0.15)	96	-6.20 (0.89)	96	NA	2	NA	4
							Placebo	98	-0.4 (0.15)	98	-4.00 (0.80)	98	NA	2	NA	5
29 Yan Gao 2009 (48)	55 (9.0)	466	207	8.3 (1.0)	16	Metformin ±SU	Exenatide 10μg	234	-1.2 (0.10)	234	-1.20 (0.30)	234	134	3	83	23
							Placebo	232	-0.4 (0.10)	232	-0.10 (0.20)	232	84	4	21	3
30 David M Kendall 2005 (49)	55 (10.0)	488	281	8.5 (1.0)	30	Metformin ±SU	Exenatide 10μg	241	-0.8 (0.10)	241	-1.60 (0.20)	241	NA	12	67	22
							Placebo	247	+0.2 (0.10)	247	-0.90 (0.20)	247	NA	15	31	11
31 Ralph A DeFronzo 2005 (50)	52 (11.0)	226	135	8.2 (1.0)	30	Metformin	Exenatide 10μg	113	-0.8 (0.10)	113	-2.8 (0.50)	113	NA	3	6	8
							Placebo	113	+0.1 (0.10)	113	-0.30 (0.30)	113	NA	4	6	1
32 John B Buse 2013 (51)	57 (9.6)	911	499	8.5 (1.0)	26	Metformin ±SU	Exenatide 2.0mg	390	-1.28 (0.05)	404	-2.68 (0.18)	461	283	13	51	12
							Liraglutide 1.8mg	386	-1.48 (0.05)	398	-3.57 (0.18)	450	307	7	40	24
33 Kishore M Gadde 2017 (52)	53.4 (9.8)	242	126	8.4 (1.0)	28	Metformin	Exenatide 2.0mg	181	-1.13 (0.11)	181	-1.12 (0.26)	181	101	5	4	3
							Placebo	61	-0.40 (0.19)	61	0.15 (0.48)	61	29	2	2	3
34 Takashi Kadowaki 2009 (53)	57.8 (10.4)	78	53	7.9 (0.9)	12	Metformin ±SU	Exenatide 10μg	37	-1.4 (0.10)	37	-1.30 (0.30)	37	35	0	20	5
							Placebo	40	0.02 (0.10)	40	-0.70 (0.20)	40	26	0	4	1

N_total_, The total number of people included in the study; N_male_, total number of males; N_1_, total number of decreased HbA1c (%); N_2_, total number of weight loss; N_3_, total number of safeties; N_T_, total number of total adverse events; N_S_, total number of serious adverse events; N_H_, Hypoglycemic events; N_AE_, total number of withdrew due to adverse events; SU, Sulfonylureas; TZD, Thiazolidinedione;. OAD, Oral Anti-Diabetic; SLGT-2, sodium-dependent glucose transporters 2.

### 3.2 Quality assessment of included study

**Figure 2:**
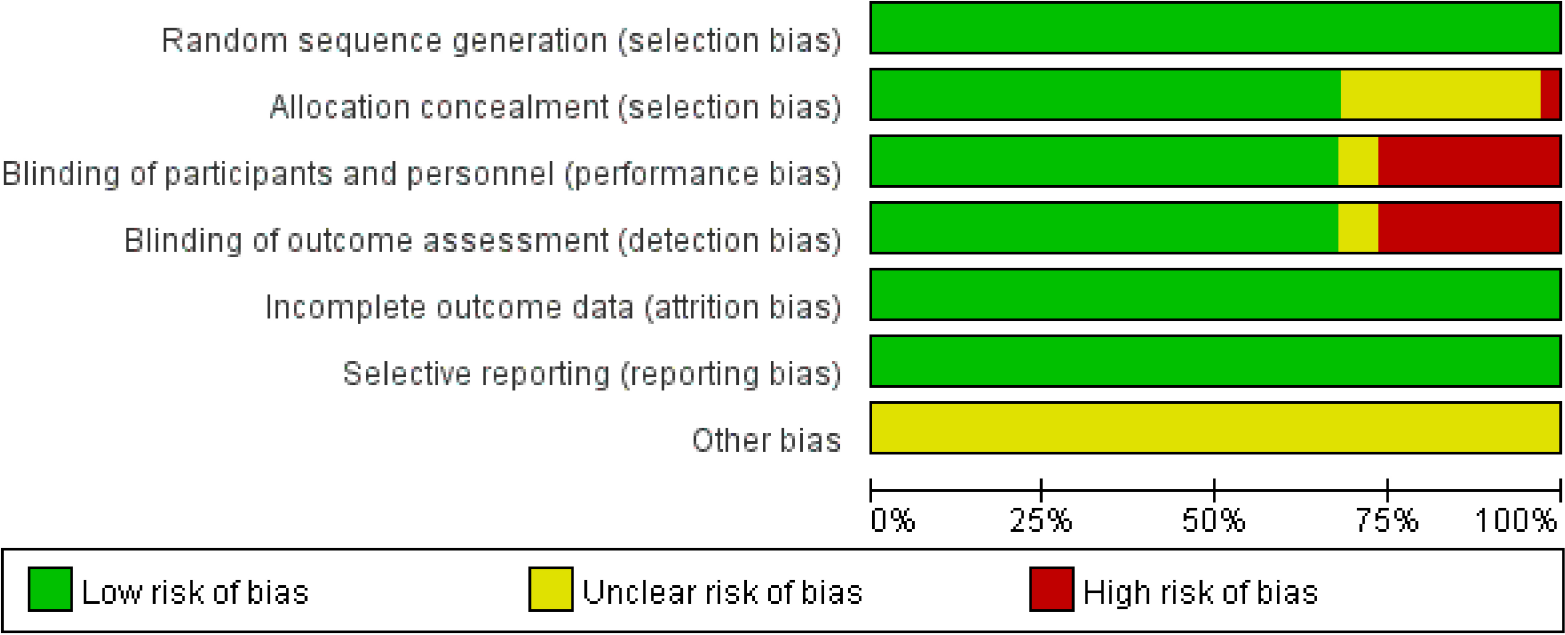
Risk of bias assessment plots.

**Figure 3:**
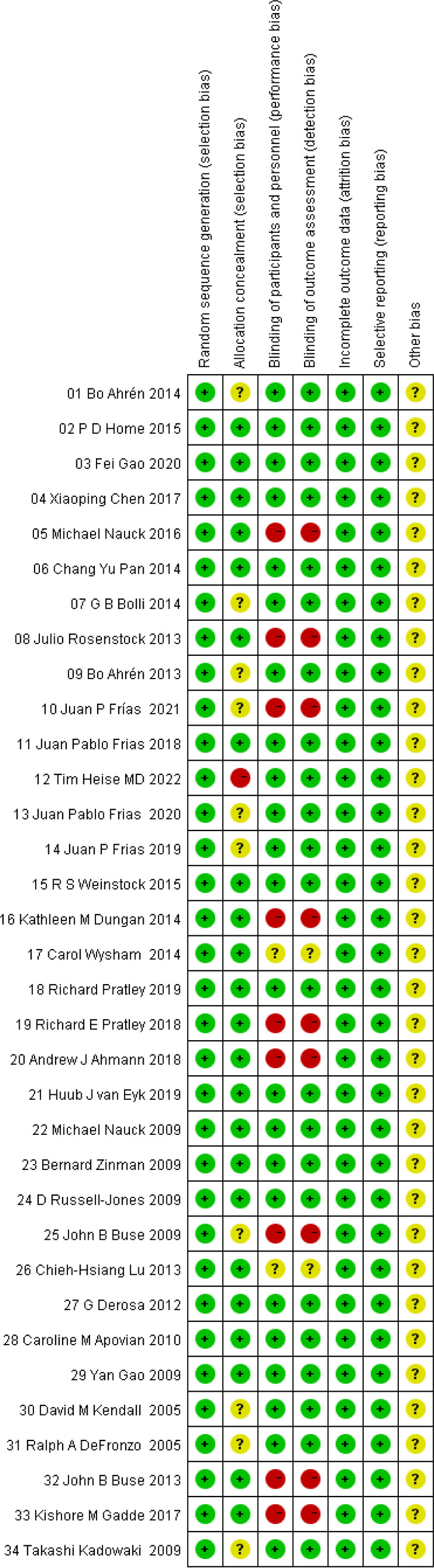
Risk of bias summary plots.

The risk of bias assessment plots and risk summary plots are shown in **Figures 2**, **3**. Regarding the risk of bias, in the 34 included studies, all studies described in detail random sequence generation, incomplete outcome data, selective reporting, and the fact that they were all considered low-risk studies. In the allocation concealment bias, 10 (29.4%) studies and 1 (2.9%) study were classified as unclear and high risk because they were not reported, and an open random allocation table was used. Regarding blinding of patients and personnel and blinding of outcome assessment, 11 (32.4%) were considered high risk because they were not blinded, and 2 (5.9%) were regarded as unclear risk because they were not reported in the study. In other biases, all studies were not reported and were considered of unclear risk.

### 3.3 Results of network meta-analysis

**Figure 4:**
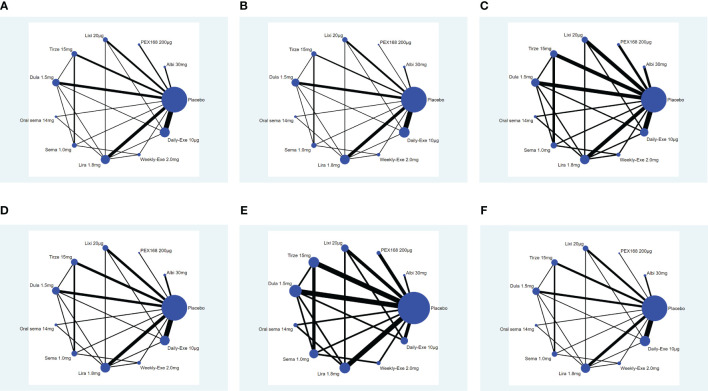
Network plot. **(A)** (Decreased HbA1c), **(B)** (Weight loss), **(C)** (The rate of adverse events), **(D)** (The rate of serious adverse events), **(E)** (Hypoglycemic events); **(F)** (AE withdrawal); (Note: Each node represents a specific intervention, the size of the nodes corresponds to the number of participants assigned to each treatment).

The network plot is shown in **Figure 4**. In the 34 included studies, 34 (100%) studies with 12993 (100%) participants reported a reduction in HbA1c, 32 (94%) studies with 12906 (99%) for weight loss, 27 (79%) studies with 11608 (89%) for the rate of adverse events, 29 (85%) studies with 12558 (96%) for the rate of serious adverse events, 30 (88%) studies with 12522 (96%) for the hypoglycemic events, 30 (88%) studies with 12638 (97%) for the rate of withdrawals due to adverse events. Each evidence network has direct and indirect comparisons, with 15 closed loops for all outcome indicators except hypoglycemia, which has 10 closed loops.

### 3.4 Result of the inconsistency analysis

The results of the global inconsistency test are shown in **Supplementary File 3** (**Table 1**). The p-values of the global inconsistency test results for the six outcome indicators of HbA1c reduction, weight loss, total adverse events, serious adverse events, hypoglycemic events, and AE withdrawal were 0.99, 0.96, 0.52, 0.28, 0.48, and 0.80, respectively, indicating that there was no inconsistency. In the local inconsistency test (node splitting method), there was inconsistency between the semaglutide 1.0 mg group and the placebo group for weight loss and AE withdrawal, and there were no significant inconsistent differences between the other group comparisons. (p ≥ 0.05).

In the loop inconsistency analysis, there were four loops for decreased HbA1c and weight loss outcome indicators and one loop of inconsistency for serious adverse events; other closed loops are not significantly inconsistent (p ≥0.05 or CI_95 include 0). The heterogeneity of each outcome indicator was calculated using I-squared; the results of the heterogeneity and loop inconsistency tests are shown in **Supplementary File 4**.

### 3.5 Decreased HbA1c (%)

The results of HbA1c reduction for 10 interventions are shown in **Table 2**. Compared with placebo, all 10 interventions were effective in reducing HbA1c (e.g., tirzepatide 15mg (MD=-2.23%, 95%_CI [-2.45, -2.01])). All 10 GLP-1RAs were significantly more effective than placebo in reducing HbA1c (p≥0.05), and tirzepatide 15mg and semaglutide 1.0mg were significantly more effective than the other GLP-1RAs.

**Table 2:** Comparisons for the Δ HbA1c (%) of the 10 Interventions.

Tirze15mg										
-0.66 (-0.91, -0.41) *	Sema1.0mg									
-1.11 (-1.49, -0.73) *	-0.45 (-0.84, -0.06) *	PEX168200μg								
-1.15 (-1.39, -0.90) *	-0.48 (-0.74, -0.23) *	-0.03 (-0.39,0.32)	Dula1.5mg							
-1.13 (-1.58, -0.68) *	-0.47 (-0.93, -0.01) *	-0.02 (-0.52, 0.48)	0.02 (-0.41, 0.45)	Oralsema14mg						
-1.23 (-1.49, -0.96) *	-0.57 (-0.84, -0.29) *	-0.12 (-0.46, 0.23)	-0.08 (-0.30, 0.13)	-0.10 (-0.50, 0.30)	Lira1.8mg					
-1.34 (-1.71, -0.97) *	-0.68 (-1.06, -0.30) *	-0.23 (-0.66, 0.20)	-0.20 (-0.54, 0.15)	-0.21 (-0.71, 0.29)	-0.11 (-0.45,0.23)	Albi30mg				
-1.41 (-1.73, -1.09) *	-0.75 (-1.04, -0.45) *	-0.30 (-0.71, 0.11) *	-0.26 (-0.56, 0.04)	-0.28 (-0.75, 0.19)	-0.18 (-0.46,0.09)	-0.07 (-0.47, 0.33)	Weekly-Exe2.0mg			
-1.44 (-1.70, -1.18) *	-0.78 (-1.05, -0.51) *	-0.33 (-0.67, 0.01)	-0.30 (-0.51, -0.08)	-0.31 (-0.73, 0.11)	-0.21 (-0.41, -0.02) *	-0.10 (-0.43, 0.23)	-0.03 (-0.33,0.26)	Daily-Exe10μg		
-1.77 (-2.07, -1.48) *	-1.11 (-1.42, -0.80) *	-0.66 (-1.03, -0.29) *	-0.63 (-0.88, -0.37) *	-0.64 (-1.08, -0.20) *	-0.54 (-0.78, -0.31) *	-0.43 (-0.79, -0.07) *	-0.36 (-0.69, -0.04) *	-0.33 (-0.55, -0.11) *	Lixi20μg	
-2.23 (-2.45, -2.01) *	-1.57 (-1.81, -1.33) *	-1.12 (-1.43, -0.81) *	-1.09 (-1.26, -0.91) *	-1.10 (-1.50, -0.70) *	-1.00 (-1.16, -0.85) *	-0.89 (-1.19, -0.59) *	-0.82 (-1.09, -0.56) *	-0.79 (-0.93, -0.65) *	-0.46 (-0.66, -0.26) *	Placebo

*Significant difference (P < 0.05).

**Figure 5:**
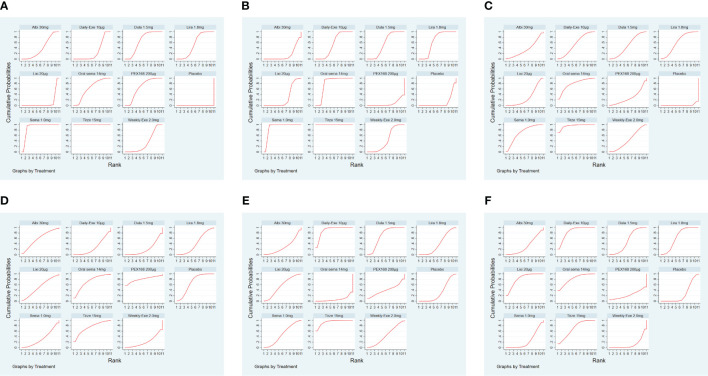
The ranking of GLP-1RA based on cumulative probability plots and SUCRA. **(A)** (Decreased HbA1c), **(B)** (Weight loss), **(C)** (The rate of adverse events), **(D)** (The rate of serious adverse events), **(E)** (Hypoglycemic events), **(F)** (AE withdrawal).

The results of SUCRA of 10 interventions and cumulative probability plots are shown in **Table 3** and **Figure 5**. According to the SUCRA results, the ranking of HbA1c reduction from highest to lowest was as follows: tirzepatide15mg>semaglutide1.0mg>PEX168-200μg>dulaglutide1.5mg>oral semaglutide14mg>liraglutide1.8mg>albiglutide30mg>weekly-exenatide2.0mg>daily-exenatide10μg>lixisenatide20μg>placebo.

**Table 3:** The SUCRA (%) results of network meta of the 6 outcome indicators.

Treatment	Decreased HbA1c	Weight Loss	Total Adverse Events	Serious Adverse Events	Hypoglycemic Events	AE withdraw
Placebo	0	11.4	1.5	67.8	32	25.3
Albi 30mg	40.3	14.9	35.4	58.8	30	31.0
PEX168 200μg	66	7.2	28.9	81.5	40.2	22.7
Lixi 20μg	10.3	28.9	29.1	53.1	55.7	82.1
Tirze 15mg	100	100	95.3	73.8	93.7	70.1
Dula 1.5mg	65.2	56.1	50	20.3	69.3	51.2
Oral sema 14mg	62.7	79.5	78.2	71.9	6.8	82.2
Sema 1.0mg	89.7	89.9	71.6	33.2	48.9	24.6
Lira 1.8mg	54.3	64.3	61.7	41.4	39.2	63.6
Weekly-Exe 2.0mg	33.7	43.4	44.1	21.3	44.4	12.1
Daily-Exe 10μg	27.8	54.4	54.1	26.9	89.8	85.1

For efficacy outcome indicators, larger SUCRA values indicated better efficacy. For safety outcome indicators, larger SUCRA values indicated a higher incidence of adverse effects and poorer results.

### 3.6 Weight loss (kg)

The weight loss results for 10 interventions are shown in **Table 4**. Compared with placebo, all 10 interventions were associated with weight loss (e.g., tirzepatide15mg (MD=-10.93kg, 95%_CI [-11.89, -9.98]). Compared with the placebo, the other 8 GLP-1RAs were more effective in reducing weight (kg), except for albiglutide 30 mg and PEX-200 μg, which did not show significant changes in weight reduction. In addition, tirzepatide 15 mg and semaglutide 1.0 mg were significantly more effective than the other interventions in reducing weight.

**Table 4:** Comparisons for the weight loss of the 10 interventions.

Tirze15mg										
-5.35 (-6.29, -4.40) *	Sema1.0mg									
-7.39 (-9.15, -5.62) *	-2.04 (-3.78, -0.30) *	Oralsema14mg								
-8.83 (-9.90, -7.75) *	-3.48 (-4.50, -2.46) *	-1.44 (2.94, 0.06)	Lira1.8mg							
-9.07 (-10.07, -8.06) *	-3.72 (-4.67, -2.78) *	-1.68 (-3.29, -0.07) *	-0.24 (-1.02,0.54)	Dula1.5mg						
-9.11 (-10.17, -8.05) *	-3.76 (-4.79, -2.74) *	-1.72 (-3.29, -0.16) *	-0.28 (-0.99,0.42)	-0.04 (-0.81, 0.73)	Daily-Exe10μg					
-9.51 (-10.75, -8.27) *	-4.17 (-5.25, -3.08) *	-2.12 (-3.87, -0.38) *	-0.69 (-1.69,0.32)	-0.44 (-1.53, 0.65)	-0.40 (-1.47, 0.67)	Weekly-Exe2.0mg				
-10.17 (-11.36, -8.98) *	-4.83 (-5.98, -3.67) *	-2.78 (-4.43, -1.14) *	-1.35 (-2.20, -0.49) *	-1.10 (-2.05, -0.15) *	-1.06 (-1.87, -0.25)	-0.66 (-1.85, 0.53)	Lixi20μg			
-10.82 (-12.27, -9.37) *	-5.47 (-6.90, -4.05) *	-3.43 (-5.29, -1.58) *	-1.99 (-3.23, -0.76) *	-1.75 (-3.02, -0.48) *	-1.71 (-2.91, -0.51) *	-1.31 (-2.77, 0.15)	-0.65 (-1.96, 0.66)	Albi30mg		
-10.93 (-11.89, -9.98) *	-5.59 (-6.50, -4.67) *	-3.55 (-5.05, -2.05) *	-2.11 (-2.69, -1.53) *	-1.87 (-2.51, -1.22) *	-1.82 (-2.32, -1.33) *	-1.42 (-2.39, -0.46) *	-0.76 (-1.49, -0.04) *	-0.11 (-1.21, 0.98)	Placebo	
-11.33 (-13.15, -9.52) *	-5.99 (-7.78, -4.19) *	-3.95 (-6.10, -1.79) *	-2.51 (-4.16, -0.86) *	-2.27 (-3.94, -0.59) *	-2.22 (-3.85, -0.60) *	-1.82 (-3.64, -0.00)	-1.16 (-2.87,0.54)	-0.51 (-2.41, 1.38)	-0.40 (-1.94, 1.14)	PEX168200μg

*Significant difference (P < 0.05).

As shown in **Table 3** and **Figure 5**, the SUCRA results show that the ranking of weight loss from highest to lowest was as follows: Tirzepatide15mg>Semaglutide1.0mg>Oral semaglutide14mg>Liraglutide1.8mg>Dulaglutide1.5mg>Daily exenatide10μg>Weekly exenatide2.0mg>Lixisenatide20μg>Albiglutide30mg>Placebo>PEX168-200μg.

### 3.7 The rate of adverse events

The results of the adverse events rate for 10 interventions are shown in **Table 5**. Tirzepatide 15 mg, oral semaglutide 14 mg, and semaglutide 1.0 mg did not differ significantly from placebo in the rate of adverse events, while the other seven GLP-1RAs did. Semaglutide1.0mg, tirzepatide15mg had a significantly higher rate of adverse events compared with the other GLP-1RAs, and oral semaglutide14mg had a significantly higher rate of adverse events compared with weekly exenatide2.0mg, albiglutide30mg, lixisenatide20μg, and PEX168-200μg. while all other head-to-head comparisons between GLP-1RAs were not significantly higher in terms of adverse event rates.

**Table 5:** The results of the rate of adverse events for 10 interventions.

Tirze15mg										
0.25 (-0.50,1.01)	Oralsema14mg									
0.37 (-0.08,0.82) *	0.11 (-0.59,0.81) *	Sema1.0mg								
0.47 (-0.08,1.01)	0.21 (-0.34,0.77) *	0.10 (-0.36,0.56) *	Lira1.8mg							
0.52 (-0.06,1.09)	0.26 (-0.36,0.88) *	0.15 (-0.35,0.65) *	0.05 (-0.30,0.39)	Daily-Exe10μg						
0.55 (0.02,1.08)	0.30 (-0.33,0.92) *	0.18 (-0.25,0.61) *	0.08 (-0.27,0.43) *	0.03 (-0.35,0.42) *	Dula1.5mg					
0.60 (0.03,1.17)	0.34 (-0.32,1.01)	0.23 (-0.21,0.68) *	0.13 (-0.27,0.53) *	0.08 (-0.40,0.56) *	0.05 (-0.41,0.50) *	Weekly-Exe2.0mg				
0.70 (-0.02,1.42)	0.44 (-0.31,1.20)	0.33 (-0.34,1.00)	0.23 (-0.34,0.80) *	0.18 (-0.40,0.77) *	0.15 (-0.45,0.75) *	0.10 (-0.55,0.75) *	Albi30mg			
0.72 (0.14,1.30)	0.46 (-0.15,1.08)	0.35 (-0.16,0.86) *	0.25 (-0.09,0.59) *	0.20 (-0.15,0.56) *	0.17 (-0.24,0.58) *	0.12 (-0.36,0.60) *	0.02 (-0.56,0.60) *	Lixi20μg		
0.77 (0.03,1.51)	0.52 (-0.27,1.30)	0.40 (-0.29,1.10)	0.30 (-0.30,0.91) *	0.25 (-0.37,0.87) *	0.22 (-0.41,0.85) *	0.17 (-0.51,0.85) *	0.07 (-0.67,0.81) *	0.05 (-0.56,0.67) *	PEX168200μg	
1.13 (0.61,1.65)	0.88 (0.31,1.44)	0.76 (0.32,1.21)	0.66 (0.40,0.93) *	0.61 (0.31,0.92) *	0.58 (0.25,0.91) *	0.53 (0.12,0.94) *	0.43 (-0.07,0.93) *	0.41 (0.13,0.70) *	0.36 (-0.18,0.90) *	Placebo

*Significant difference (P < 0.05).

As shown in **Table 3** and **Figure 5**, the SUCRA results show that the ranking of the rate of adverse events from highest to lowest was as follows: Tirzepatide15mg>Oral semaglutide14mg>Semaglutide1.0mg>Liraglutide1.8mg>Daily exenatide10μg>Dulaglutide1.5mg>Weekly exenatide2. 0mg>Albiglutide30mg>Lixisenatide20μg>PEX168-200μg>Placebo, this result indicates that tirzepatide15mg had the highest incidence of adverse events compared to all other interventions.

### 3.8 The rate of serious adverse events

The results of the serious adverse event rate for 10 interventions are shown in **Table 6**. Compared to the placebo, there was a significant difference in the incidence of serious adverse events between tirzepatide 15 mg and oral semaglutide 14 mg. In addition, the SUCRA values for PEX 168-200 μg, tirzepatide 15 mg, and oral semaglutide 14 mg were higher than placebo, meaning that PEX 168-200 μg, tirzepatide 15 mg and oral semaglutide 14 mg had the highest incidence of serious adverse events compared to placebo.

**Table 6:** The results of the rate of serious adverse events for 10 interventions.

PEX16820μg										
0.39 (-1.29, 2.08)	Tirze15mg									
0.45 (-1.13, 2.03)	0.06 (-0.94,1.05)	Oralsema14mg								
0.54 (-0.92, 2.01)	0.15 (-0.68,0.99) *	0.09 (-0.49,0.68) *	Placebo							
0.59 (-0.98, 2.16)	0.19 (-0.82,1.21)	0.14 (-0.68,0.95) *	0.04 (-0.53,0.61) *	Albi30mg						
0.67 (-0.90, 2.24)	0.27 (-0.72,1.27)	0.22 (-0.55,0.99) *	0.12 (-0.43,0.68) *	0.08 (-0.71,0.88) *	Lixi20μg					
0.78 (-0.73, 2.29)	0.39 (-0.48,1.26)	0.33 (-0.23,0.89) *	0.24 (-0.13,0.60) *	0.19 (-0.48,0.87) *	0.11 (-0.48,0.70) *	Lira1.8mg				
0.90 (-0.70, 2.49)	0.50 (-0.15, 1.15)	0.45 (-0.37,1.26)	0.35 (-0.28,0.98) *	0.31 (-0.54,1.16)	0.23 (-0.59,1.04)	0.11 (-0.54,0.76) *	Sema1.0mg			
0.93 (-0.59, 2.45)	0.53 (-0.36, 1.43)	0.48 (-0.21,1.16)	0.38 (-0.03,0.80) *	0.34 (-0.36,1.04)	0.26 (-0.35,0.87) *	0.15 (-0.34,0.63) *	0.03 (-0.66,0.73) *	Daily-Exe10μg		
1.04 (-0.58, 2.66)	0.65 (-0.27, 1.56)	0.59 (-0.26,1.44)	0.50 (-0.19,1.18)	0.45 (-0.44,1.34)	0.37 (-0.46,1.21)	0.26 (-0.41,0.93) *	0.15 (-0.45,0.74) *	0.11 (-0.64,0.87) *	Weekly-Exe2.0mg	
1.01 (-0.54, 2.57)	0.62 (-0.17, 1.40)	0.56 (-0.18,1.30)	0.47 (-0.05,0.98) *	0.43 (-0.34,1.19)	0.34 (-0.38,1.07)	0.23 (-0.32,0.79) *	0.12 (-0.42,0.66) *	0.09 (-0.49,0.66) *	-0.03 (-0.73,0.68) *	Dula1.5mgs

*Significant difference (P < 0.05).

As shown in **Table 3** and **Figure 5**, the SUCRA results suggest that the ranking of the rate of serious adverse events from high to low was as follows: PEX168-200μg>Tirzepatide15mg>Oralsemaglutide14mg>Placebo>Albiglutide30mg>Lixisenatide20μg>Liraglutide1.8mg>Semaglutide1.0mg>Daily-Exenatide10μg>Weekly-Exenatide2. 0mg>Dulaglutide1.5mg, it means that PEX168-200μg had the highest incidence of serious adverse events compared to all other interventions.

### 3.9 Hypoglycemic events

The results of hypoglycemic events for 5 interventions including sulfonylureas, and 10 interventions excluding sulfonylureas are shown in **Tables 7**, **8**. There was no significant difference in hypoglycemic events when sulfonylureas were included in combination therapy with the 5 GLP-1RAs compared with placebo. There was no significant difference in hypoglycemic events in head-to-head comparisons. When sulfonylureas were not included among the drugs in combination therapy, there was a higher rate of hypoglycemic events with semaglutide 1.0 mg versus exenatide 2.0 mg and liraglutide 1.8 mg versus placebo, and no significant difference of hypoglycemic events in the other head-to-head comparisons. However, the hypoglycemic events (OR value) were significantly higher when the combination therapy included a sulfonylurea than when there was no sulfonylurea, suggesting that sulfonylureas can dramatically increase the incidence of hypoglycemia events. Meanwhile, there was no significant difference in the incidence of hypoglycemic events with or without sulfonylureas compared to placebo, suggesting that GLP-1RAs do not increase the incidence of hypoglycemic events.

**Table 7:** The results of the hypoglycemic events for 5 interventions include sulfonylurea drugs.

Daily-Exe10μg					
0.37 (-0.82,1.56)	Weekly-Exe2.0mg				
0.61 (-0.13,1.35)	0.24 (-0.69,1.17)	Lira1.8mg			
0.71 (-0.78,2.20)	0.34 (-1.46,2.14)	0.10 (-1.45,1.64)	Lixi20μg		
0.78 (-0.43,2.00)	0.41 (-1.17,2.00)	0.17 (-1.11,1.45)	0.07 (-1.64,1.78)	Albi30mg	
1.52 (0.89,2.14)	1.15 (-0.04,2.34)	s0.91 (0.16,1.65)	0.81 (-0.54,2.16)	0.74 (-0.31,1.78)	Placebo

*Significant difference (P < 0.05).

**Table 8:** The results of the hypoglycemic events for 10 interventions exclude sulfonylurea drugs.

Tirze15mg										
0.24 (-0.84,1.32)	Daily-Exe10μg									
0.77 (-0.25,1.79)	0.53 (0.02,1.03)	Dula1.5mg								
0.99 (-0.28,2.26)	0.74 (-0.05,1.53)	0.21 (-0.66,1.09)	Lixi20μg							
1.14 (0.16,2.13)	0.90 (-0.10,1.90)	0.37 (-0.55,1.29)	0.16 (-1.05,1.36)	Sema1.0mg						
1.21 (0.10,2.33)	0.97 (-0.13,2.06)	0.44 (-0.59,1.46)	0.22 (-1.13,1.57)	0.07 (-0.52,0.65) *	Weekly-Exe2.0mg					
1.42 (-1.08,3.91)	1.17 (-1.18,3.53)	0.64 (-1.70,2.99)	0.43 (-2.02,2.87)	0.27 (-2.19,2.74)	0.21 (-2.29,2.71)	PEX168200μg				
1.30 (0.22,2.38)	1.06 (0.41,1.70)	0.53 (-0.02,1.07)	0.31 (-0.59,1.21)	0.16 (-0.85,1.16)	0.09 (-1.00,1.18)	-0.12 (-2.46,2.23)	Lira1.8mg			
1.41 (0.41,2.42)	1.17 (0.59,1.75)	0.64 (0.08,1.20)	0.43 (-0.45,1.31)	0.27 (-0.67,1.21)	0.20 (-0.82,1.23)	-0.00 (-2.28,2.28)	0.12 (-0.43,0.66) *	Placebo		
1.56 (0.08,3.04)	1.32 (0.08,2.55)	0.79 (-0.44,2.01)	0.57 (-0.83,1.97)	0.42 (-1.02,1.86)	0.35 (-1.14,1.84)	0.14 (-2.38,2.67)	0.26 (-0.96,1.48)	0.15 (-0.94,1.24)	Albi30mg	
2.56 (0.71,4.41)	2.32 (0.67,3.96)	1.79 (0.17,3.41)	1.57 (-0.19,3.34)	1.42 (-0.40,3.23)	1.35 (-0.51,3.21)	1.14 (-1.64,3.92)	1.26 (-0.29,2.81)	1.15 (-0.44,2.73)	1.00 (-0.93,2.93)	Oralsema14mg

*Significant difference (P < 0.05).

As shown in **Table 3** and **Figure 5**, the SUCRA results indicated that the ranking of the hypoglycemic events from high to low excluding sulfonylureas as follows: Tirzepatide15mg>Daily-Exenatide10μg>Dulaglutide1.5mg>Lixisenatide20μg>Semaglutide1.0mg>Weekly-Exenatide2.0mg>PEX168-200μg>Liraglutide1. 8mg>Placebo>Albiglutide30mg>Oral semaglutide14mg, and it suggested that tirzepatide15mg had the highest incidence of hypoglycemic events compared with all other GLP-1RAs.

### 3.10 AE withdrawal

The results of the AE discontinuation rate for 10 interventions are shown in **Table 9**. Compared to the placebo, the other eight GLP-1RAs were similar regarding adverse event withdrawal rates, except for a significant difference between albiglutide 30 mg and semaglutide 1.0 mg. Semaglutide 1.0 mg versus exenatide 2.0 mg, oral semaglutide 14 mg versus exenatide 10 μg, and lixisenatide 20 μg showed significant differences in adverse event withdrawal rates. Lixisenatide 20 μg significantly differed from dulaglutide 1.5 mg, tirzepatide 15 mg, lixisenatide 20 μg, and exenatide 10 μg. The other head-to-head comparisons between GLP-1RAs did not show a significantly higher rate of adverse event withdrawal.

**Table 9:** The results of the rate of AE withdrew for 10 interventions.

Daily-Exe10μg										
0.04 (-0.54,0.62) *	Lixi20μg									
-0.02 (-1.01,0.98) *	-0.05 (-1.09,0.98) *	Oralsema14mg								
0.26 (-0.64,1.16)	0.23 (-0.77,1.22)	0.28 (-0.94,1.50)	Tirze15mg							
0.35 (-0.17,0.87) *	0.31 (-0.29,0.91) *	0.36 (-0.51,1.24)	0.08 (-0.79,0.96) *	Lira1.8mg						
0.57 (-0.09,1.23)	0.54 (-0.24,1.31)	0.59 (-0.46,1.64)	0.31 (-0.46,1.08)	0.23 (-0.39,0.84) *	Dula1.5mg					
s0.97 (0.01,1.94)	0.93 (-0.09,1.96)	0.99 (-0.28,2.26)	0.71 (-0.48,1.89)	0.62 (-0.34,1.59)	0.40 (-0.64,1.44)	Albi30mg				
1.04 (0.58,1.50)	1.00 (0.43,1.58)	1.05 (0.11,2.00)	0.77 (-0.05,1.59)	0.69 (0.24,1.15)	0.46 (-0.13,1.06)	0.07 (-0.78,0.92) *	Placebo			
1.08 (0.26,1.91)	1.05 (0.12,1.97)	1.10 (-0.06,2.26)	0.82 (0.12,1.51)	0.73 (-0.04,1.51)	0.51 (-0.16,1.17)	0.11 (-1.02,1.24)	0.04 (-0.70,0.79) *	Sema1.0mg		
1.43 (-0.54,3.40)	1.39 (-0.61,3.39)	1.44 (-0.70,3.58)	1.16 (-0.92,3.25)	1.08 (-0.89,3.05)	0.85 (-1.16,2.86)	0.46 (-1.64,2.55)	0.39 (-1.53,2.31)	0.35 (-1.71,2.40)	PEX168200μg	
1.36 (0.54,2.19)	1.32 (0.41,2.24)	1.38 (0.24,2.52)	1.10 (0.21,1.99)	1.01 (0.27,1.75)	0.79 (0.01,1.56)	0.39 (-0.75,1.53)	0.32 (-0.43,1.08)	0.28 (-0.41,0.97) *	-0.07 (-2.13,1.99)	Weekly-Exe2.0mg

*Significant difference (P < 0.05).

As shown in **Table 3** and **Figure 5**, the SUCRA results indicate that the ranking of AE withdrawals from highest to lowest was as follows: Daily exenatide10μg>Lixisenatide20μg>Oral semaglutide14mg>Tirzepatide15mg>Liraglutide1.8mg>Dulaglutide1.5mg>Albiglutide30mg>Placebo>Semaglutide1. 0mg>PEX168-200μg>weekly-exenatide2.0mg, it suggested that daily-exenatide10μg had the highest incidence of AE withdrawal compared to all other interventions.

### 3.11 Cluster analysis

Cluster analysis was performed based on the SUCRA values for clinical efficacy (reduction in HbA1c) and safety (the rate of adverse events), and the results of the cluster analysis are shown in **Supplementary File 3** (**Figure 1**). Compared with other interventions, 15 mg of tirzeptide had a significant advantage in efficacy, but it had a higher rate of adverse events than other GLP-1RAs. Semaglutide1.0mg, oral semaglutide14mg, liraglutide1.8mg, dulaglutide1.5mg, PEX168-200μg, albiglutide30mg, daily exenatide10μg, weekly exenatide2.0mg have a similar advantage in terms of efficacy and safety. Lixisenatide 20 µg has an efficacy advantage but not in security (the rate of adverse events).

### 3.12 Sensitivity analysis

**Supplementary File 3** (**Table 2**) shows the sensitivity analysis results. Stata software was used to perform sensitivity analyses for the primary outcome indicator (HbA1c reduction) after excluding 10 high-risk studies (5, 8, 10, 12, 16, 19, 20, 25, 32, 33) according to results 3.2 Quality assessment of included studies. **Supplementary File 3** (**Table 2**) shows that the network meta-analysis did not change significantly without inversion, indicating that the results were reliable.

### 3.13 Publication bias analysis

**Figure 6:**
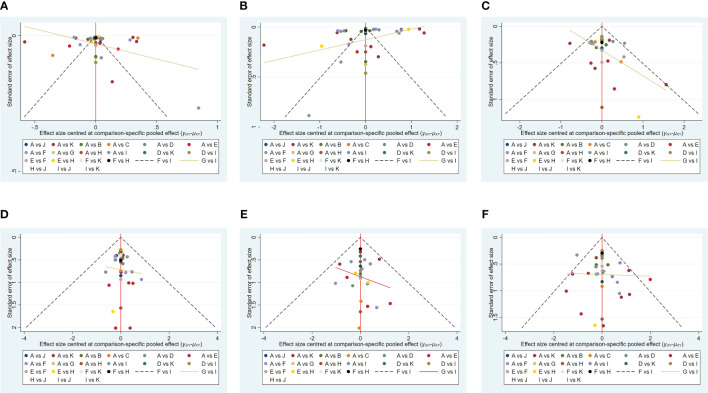
The funnel plots for efficacy and safety. **(A)** (Decreased HbA1c), **(B)** (Weight loss), **(C)** (The rate of adverse events), **(D)** (The rate of serious adverse events), **(E)** (Hypoglycemic events), **(F)** (AE withdrawal), (Note: A: Placebo; B: Albi30mg; C: PEX168-200μg; D: Lixisenatide20μg; E: Tirze15mg; F: Dulaglutide1.5mg; G: Oralsema14mg; H: Sema1.0mg; I: Lira1.8mg; J: Weekly-Exenatide2.0mg; K: Daily-Exenatide10μg).

Stata software generated funnel plots for six outcome measures: HbA1c reduction, weight loss, total adverse events, serious adverse events, hypoglycemic events, and AE discontinuation. The funnel plots for efficacy and safety are shown in **Figure 6**. **Figure 6** shows that the distribution of studies in the funnel plot is approximately symmetric, suggesting no publication or other bias between studies. However, in **Figures 6A, B**, there is heterogeneity in some studies with scatter plots outside the funnel plot, possibly due to minor sample effects.

## 4 Discussion

This review was based on 34 RCTs involving 12,993 patients with type 2 diabetes who had poor glycemic control on metformin and were randomized to 10 GLP-1RAs or placebo. In the 34 included studies, 18 used metformin + GLP-1RA as the therapeutic agent, and 16 used metformin + GLP-1RA ± OAD (e.g., sulfonylurea, thiazolidinedione, and sodium-glucose cotransporter-2 inhibitors). According to the NMA results, after treatment (82.4%≥ 24 weeks), all GLP-1RAs were superior to placebo in efficacy. In terms of HbA1c reduction, the most effective drug was tirzepatide 15 mg (-2.23%), followed by semaglutide 1.0 mg (-1.57%) and PEX 168200 (-1.12%). In terms of weight loss, tirzepatide 15 mg was the best (-11.3 kg), followed by semaglutide 1.0 mg (-5.99 kg) and oral semaglutide 14 mg (-3.95 kg). This result suggests that tirzepatide 15mg and semaglutide 1.0mg have a clear advantage in reducing blood glucose and weight in patients with T2D.

In terms of safety, tirzepatide 15mg (OR=1.13), oral semaglutide 14mg (OR=0.88), and semaglutide 1.0mg (OR=0.76) were more frequent than the other GLP-1RAs in the rate of adverse events (e.g., nausea, vomiting, and diarrhea, etc.). The OR values for tirzepatide 15mg were more significant than 1, indicating a higher risk of adverse events. In addition, tirzepatide 15mg, oral semaglutide 14mg, and semaglutide 1.0mg did not show a significantly higher incidence of adverse events. The incidence of serious adverse events (e.g., cardiovascular events, severe gastrointestinal reactions, infections, etc.) was higher for PEX168 (OR=0.54), tirzepatide 15mg (OR=0.15), and oral semaglutide 14mg (OR=0.09) than for other GLP-1RAs, and tirzepatide 15mg and oral semaglutide 14mg will have serious adverse events but at a lower risk.

GLP-1RAs generally do not cause hypoglycemia in patients with type 2 diabetes who are not taking insulin (54). However, it is noteworthy that when the combination included sulfonylureas, hypoglycemia (OR) incidence was significantly higher with GLP-1RAs, suggesting that sulfonylureas are an essential contributor to hypoglycemia (55). The 10 GLP-1RAs did not exhibit a significantly higher incidence of AE withdrawal.

Tirzepatide is a novel GIP and GLP-1 dual receptor agonist that the U.S. FDA approved for treating T2D in 2022. it has demonstrated potent HbA1c and body weight reduction. In a recently completed Phase 3, double-blind, randomized, controlled trial of once-weekly tirzepatide in the treatment of obesity, tirzepatide 15 mg achieved a mean percentage change in body weight of -20.9% (-21.9 kg) over 72 weeks of treatment, suggesting that tirzepatide 15 mg is also a potential weight loss agent (56). The result is a significant breakthrough in dual-targeted GLP-1 and GIP drugs that reduce HbA1c and body weight more effectively than GLP-1 RA. In addition, a phase 2 RCT demonstrated that retatrutide, a GIP, GLP-1, and glucagon receptor agonist achieved clinically meaningful improvements in glycemic control (2.02% reduction in HbA1c with 24 weeks of treatment) and significant weight reduction (17.18kg reduction in weight with 36 weeks of treatment), and its safety profile was consistent with that of GLP-1 receptor agonists as well as GIP and GLP-1 receptor agonists (57). In the future, GIP, GLP-1, and glucagon tri-agonists are promising for patients with T2D and/or obesity.

Semaglutide1.0mg and oral semaglutide 14mg was inferior to tirzepatide in reducing HbA1c and body weight but had significant advantages over other GLP-1RAs. In addition, oral semaglutide offers excellent convenience to patients and improves compliance. The results were generally consistent with Xia L et al. 's (58) efficacy in lowering HbA1c and body weight. However, oral semaglutide treatment increases the incidence of nausea, vomiting, and diarrhea (Comparison with placebo or other OAD (e.g., Sitagliptin, empagliflozin)), and the oral route of administration is strongly associated with an increase in gastrointestinal adverse events (59). The higher AE withdrawal rate for oral semaglutide than for subcutaneous semaglutide and tirzepatide may be related to gastrointestinal adverse events.

The global and local inconsistency test results showed no significant inconsistency for each outcome indicator and two comparison groups. In the closed loop inconsistency test, the same four closed loops showed significant inconsistency in the two outcome indicators of HbA1c reduction and weight loss. The other closed loops were those for which there was no evidence of inconsistency (p >0.05 or CI_95 includes 0). In conclusion, the results for the inconsistency of the whole network are reliable.

In sensitivity analyses, the NMA results did not change significantly and were reliable. Overall, the risk of the included studies was low, the quality was good, and the NMA results were reliable. The publication bias results suggest that there may be some bias in the effectiveness due to differences in the drugs used and the effect of a small sample size. The heterogeneity results indicate a significant difference in efficacy, and the main reason for this was the difference in the use of background medication (metformin +/-OAD). In conclusion, heterogeneity does not significantly impact the NMA results.

T2D places a heavy burden on the body, causing various complications (cardiovascular and cerebrovascular complications, microvascular and neurological lesions, diabetic foot, etc.) that can reduce the quality of life and life expectancy (60). The anti-inflammatory effects of metformin and GLP-1RAs contribute to their beneficial effects, as T2DM is characterized by chronic low-grade inflammation. In addition to glycemic control, aggressive cardiac risk reduction is a priority for all patients with T2D. Evidence suggests (61, 62) that aggressive reduction of multiple risk factors (weight loss or maintenance, smoking cessation, blood pressure control, lipid-lowering, dietary modification, and exercise) reduces the risk of microvascular and macrovascular complications in patients with diabetes.

GLP-1RAs are safe and effective and can reduce weight and the risk of major cardiovascular disease (63). Semaglutide 2.4mg and liraglutide 3.0mg have been approved by the U.S. Food and Drug Administration (FDA) for weight management in people who are overweight or obese. A related weight loss meta-analysis (15) showed that treatment with semaglutide 2.4 mg and liraglutide 3.0 mg for more than 20 weeks in combination with daily diet and exercise resulted in weight loss of 12.47 kg and 5.24 kg, respectively, in people with overweight and obesity. Tirzepatide 15 mg is expected to be approved shortly for weight control in adults with obesity or overweight (with >= 1+ obesity-related comorbidity). Liraglutide, dulaglutide, and semaglutide have been shown to have cardiovascular benefits, and the only other glucose-lowering agents for T2D that have been shown to have cardiovascular benefits are the SGLT-2 inhibitors empagliflozin, canagliflozin and dapagliflozin (64, 65). In clinical practice, selecting a GLP-1RA with cardiovascular efficacy, good glucose-lowering, and weight-loss (kg) effects, and a high safety profile based on the patient’s medical condition and needs is critical. Based on efficacy and safety results, tirzepatide 15mg, semaglutide 1.0mg, and oral semaglutide 14mg are good options for patients with T2DM.

The main objective of this study is to, directly and indirectly compare the safety and efficacy of 10 GLP-1RAs in combination with metformin, to address the wide variety of medications encountered in clinical practice, and to provide evidence-based support and reference for the use of GLP-1RAs in clinical practice. Meanwhile, being overweight and obese are risk factors for diabetes, and this study evaluated the effect of weight loss with different GLP-1RAs and glucose lowering. However, there are limitations to this study: First, the range of drug treatment cycles is vast, mostly between 12 and 48 weeks, which affects the safety assessment results. Second, in this review, there were some studies where the background drug was metformin and others where it was metformin +/- OAD, which is the source of the heterogeneity in the efficacy assessment. Third, the populations included in this study include Caucasian, African and Asian populations. However, there are some differences in the physical condition of patients with type 2 diabetes from different regions or races, and their actual clinical efficacy is also biased. Finally, there were small samples of studies and high-risk studies in the included studies, which may make our results insubstantial and incomplete. Even though there are some limitations in this study, more and more large-sample, high-quality studies will gradually emerge over time so that our evaluation results will improve and become more convincing overall.

## 5 Conclusion

This study results showed that GLP-1RAs were effective in lowering HbA1c and reducing body weight without leading to increased incidence of hypoglycemic reactions. In addition, this study may provide reference and evidence-based medical evidence for clinicians to select GLP-1RAs in patients with T2DM and high BMI. Tirzepatide 15mg, semaglutide 1.0 mg, and oral semaglutide 14 mg may be preferred for treating patients with T2DM. Finally, there is an urgent need for more high-quality, large-sample randomized controlled trials to make our study results more comprehensive and reliable.

## Author contributions

All co-authors made substantial contributions to this article and this version is published as the final version, agreeing to submit this study to the journal and taking responsibility for all aspects of the article. All authors contributed to the article and approved the submitted version.
