# Supplementary material for: Comparison of the efficacy and safety of 10 glucagon-like peptide-1 receptor agonists as add-on to metformin in patients with type 2 diabetes: a systematic review
Source: Front Endocrinol (Lausanne). 2023 Aug 28;14:1244432. doi: 10.3389/fendo.2023.1244432 (PMC10493284; doi:10.3389/fendo.2023.1244432)
Supplement: Supplementary file 1 [file DataSheet_1.zip › Supplementary Files/Table 2.docx]

**Supplementary File 2: Search Strategy**

**A=Contained one of these terms in any field:**

“Diabetes mellitus” or “type 2 diabetes” or “type II diabetes” or “non-insulin dependent diabetes” or “NIDDM” or “Diabetes Mellitus, Maturity Onset” or “Type 2 Diabetes Mellitus” or “Adult-Onset Diabetes Mellitus”

**B=Contained one of these terms in any field:**

“Glucagon like peptide 1 receptor agonist” or “glucagon-like peptide 1 receptor agonist” or “glucagon-like peptide 1 agonist” or “GLP-1 receptor agonist” or “GLP-1 agonist” or “GLP-1” or “glucagon like peptide 1” or “exenatide” or “liraglutide” or “benaglutide” or “beinaglutide” or “lixisenatide” or “dulaglutide” or “loxenatide” or “PEX168” or “semaglutide” or “tirzepatide”

**C=Contained one of these terms in any field:**

“Randomized controlled trial” or “(RCT)” or “randomised controlled trial” or “randomized trial” or “randomised trial” or “controlled clinical trial” or “randomly” or “RCT”

Finally, The PubMed, Embase, and Cochrane Library databases were used to search A and B and C, respectively.
