# Supplementary material for: Comparison of the efficacy and safety of 10 glucagon-like peptide-1 receptor agonists as add-on to metformin in patients with type 2 diabetes: a systematic review
Source: Front Endocrinol (Lausanne). 2023 Aug 28;14:1244432. doi: 10.3389/fendo.2023.1244432 (PMC10493284; doi:10.3389/fendo.2023.1244432)

**Supplementary File 3:**

Table-1 Design-by-Treatment Test

|  | Chi^2^ | Prob > chi2 |
| --- | --- | --- |
| Δ HbA1c (%) | 4.60 | 0.99 |
| Δ Weight(kg) | 5.56 | 0.96 |
| Total adverse events | 13.04 | 0.52 |
| Serious adverse events | 16.49 | 0.28 |
| Hypoglycemic episodes | 11.50 | 0.48 |
| AE withdraw | 9.39 | 0.80 |

Table 2-Comparisons for the Decreased HbA1c of the 10 Interventions After 10 High-Risk Studies Were Excluded

| Tirze15mg |  |  |  |  |  |  |  |  |
| --- | --- | --- | --- | --- | --- | --- | --- | --- |
| -1.17  (-1.67, -0.67) * | PEX168200μg |  |  |  |  |  |  |  |
| -1.21  (-1.58, -0.83) * | -0.03  (-0.47,0.40) | Dula1.5mg |  |  |  |  |  |  |
| -1.22  (-1.81, -0.64) * | -0.05  (-0.65,0.55) | -0.02  (-0.55,0.51) | Oralsema14mg |  |  |  |  |  |
| -1.35  (-1.77, -0.93) * | -0.17  (-0.61,0.26) | -0.14  (-0.48,0.20) | -0.12  (-0.60,0.35) | Lira1.8mg |  |  |  |  |
| -1.41  (-1.90, -0.92) * | -0.24  (-0.75,0.27) | -0.20  (-0.63,0.23) | -0.19  (-0.78,0.41) | -0.06  (-0.49,0.37) | Albi30mg |  |  |  |
| -1.47  (-1.85, -1.09) * | -0.30  (-0.71,0.11) | -0.27  (-0.55,0.02) | -0.25  (-0.75,0.26) | -0.13  (-0.43,0.18) | -0.06  (-0.46,0.34) | Daily-Exe10μg |  |  |
| -1.88  (-2.33, -1.43) * | -0.71  (-1.18, -0.24) * | -0.68  (-1.06, -0.30) * | -0.66  (-1.21, -0.10) * | -0.54  (-0.92, -0.16) * | -0.47  (-0.93, -0.01) * | -0.41  (-0.75, -0.07) * | Lixi20μg |  |
| -2.30  (-2.64, -1.96) * | -1.13  (-1.49, -0.76) * | -1.09  (-1.33, -0.85) * | -1.08  (-1.55, -0.60) * | -0.95  (-1.20, -0.71) * | -0.89  (-1.25, -0.53) * | -0.83  (-1.01, -0.65) * | -0.42  (-0.71, -0.13) * | Placebo |

Note: *Significant difference (P < 0.05).

**Figure 1:** The cluster analysis plot for efficacy and safety;(Efficacy= Decreased HbA1c, Safety= The rate of adverse events).


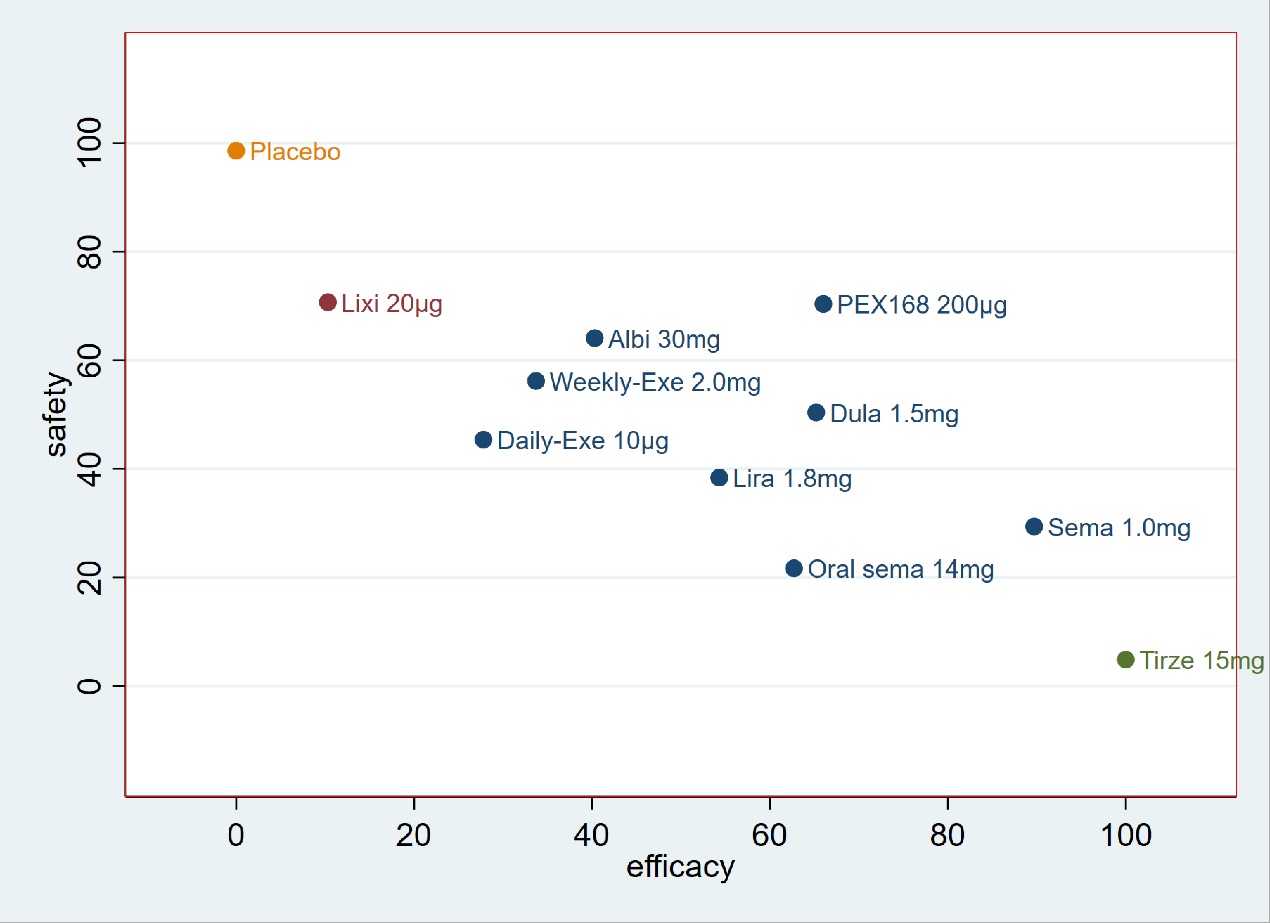

Supplement: Supplementary file 1 [file DataSheet_1.zip › Supplementary Files/Table 3.docx]
