# Supplementary material for: Comparison of the efficacy and safety of 10 glucagon-like peptide-1 receptor agonists as add-on to metformin in patients with type 2 diabetes: a systematic review
Source: Front Endocrinol (Lausanne). 2023 Aug 28;14:1244432. doi: 10.3389/fendo.2023.1244432 (PMC10493284; doi:10.3389/fendo.2023.1244432)
Supplement: Supplementary file 1 [file DataSheet_1.zip › Supplementary Files/Table 4.docx]

**Supplementary File 4:**

**4.1 Pairwise meta-analysis**

**4.1.1 Efficacy**

**Δ HbA1c (%)**

| Comparisons | No. of studies | P-value | I^2^ | Chi2 |
| --- | --- | --- | --- | --- |
| Albi30mg VS Placebo | 2 | 0.16 | 48% | 1.94 |
| PEX168-200μg VS Placebo | 2 | 1.00 | 0 | 0 |
| Lixi 20μg VS Placebo | 3 | 0.00001 | 96% | 53.81 |
| Tirze15mg VS Placebo | 3 | 0.00001 | 96% | 47.19 |
| Dula1.5mg VS Placebo | 3 | 0.00001 | 100% | 1035.57 |
| Lira1.8mg VS Placebo | 4 | 0.17 | 43% | 3.54 |
| Exenatide 10μg VS Placebo | 7 | 0.00001 | 98% | 274.39 |

**Note:** If p-value for Q test < 0.10 or I2 > 50% was defined as significant heterogeneity. The reason for the above heterogeneous results is due to the presence of different background therapeutic agents (Metformin ± Oral anti-diabetic drug) in each study.

**Weight loss (Kg)**

| Comparisons | No. of studies | P-value | I^2^ | Chi2 |
| --- | --- | --- | --- | --- |
| Albi30mg VS Placebo | 2 | 0.0008 | 91% | 11.23 |
| Lixi 20μg VS Placebo | 3 | 0.00001 | 99% | 280 |
| Tirze15mg VS Placebo | 2 | 0.34 | 0 | 0.91 |
| Dula1.5mg VS Placebo | 3 | 0.00001 | 100% | 531.81 |
| Lira1.8mg VS Placebo | 5 | 0.00001 | 99% | 683.37 |
| Exenatide 10μg VS Placebo | 7 | 0.00001 | 100% | 274.39 |

**Note:** If p-value for Q test < 0.10 or I2 > 50% was defined as significant heterogeneity. The reason for the above heterogeneous results is due to the presence of different background therapeutic agents (Metformin ± Oral anti-diabetic drug) in each study.

**4.1.2 Safety**

**Total adverse events**

| Comparisons | No. of studies | P-value | I^2^ | Chi2 |
| --- | --- | --- | --- | --- |
| Albi30mg VS Placebo | 2 | 0.54 | 0 | 0.38 |
| PEX168-200μg VS Placebo | 2 | 0.23 | 29% | 1.41 |
| Lixi 20μg VS Placebo | 3 | 0.20 | 38% | 3.25 |
| Tirze15mg VS Placebo | 3 | 0.78 | 0 | 0.5 |
| Dula1.5mg VS Placebo | 2 | 0.58 | 0 | 0.3 |
| Lira1.8mg VS Placebo | 3 | 0.01 | 77% | 8.54 |
| Exenatide 10μg VS Placebo | 3 | 0.19 | 40% | 3.35 |

**Serious adverse events**

| Comparisons | No. of studies | P-value | I^2^ | Chi2 |
| --- | --- | --- | --- | --- |
| Albi30mg VS Placebo | 2 | 0.84 | 0 | 0.04 |
| Lixi 20μg VS Placebo | 3 | 0.59 | 0 | 1.06 |
| Tirze15mg VS Placebo | 3 | 0.46 | 0 | 0.53 |
| Dula1.5mg VS Placebo | 2 | 0.62 | 0 | 0.25 |
| Lira1.8mg VS Placebo | 4 | 0.85 | 0 | 0.79 |
| Exenatide 10μg VS Placebo | 5 | 0.99 | 0 | 0.08 |

**Hypoglycemic events**

| Comparisons | No. of studies | P-value | I^2^ | Chi2 |
| --- | --- | --- | --- | --- |
| Lixi 20μg VS Placebo | 2 | 0.68 | 0 | 0.18 |
| Tirze15mg VS Placebo | 3 | 0.54 | 0 | 1.24 |
| Lira1.8mg VS Placebo | 3 | 0.61 | 0 | 1.00 |

**AE Withdrawal**

| Comparisons | No. of studies | P-value | I^2^ | Chi2 |
| --- | --- | --- | --- | --- |
| Albi30mg VS Placebo | 2 | 0.51 | 0 | 0.44 |
| Lixi 20μg VS Placebo | 3 | 0.76 | 0 | 0.55 |
| Tirze15mg VS Placebo | 3 | 0.02 | 75% | 8.13 |
| Dula1.5mg VS Placebo | 2 | 0.95 | 0 | 0.00 |
| Lira1.8mg VS Placebo | 5 | 0.08 | 55% | 6.66 |
| Exenatide 10μg VS Placebo | 6 | 0.13 | 42% | 8.60 |

**Supplementary File 4.2:**

**4.2.1 The results of loop inconsistencies test**

The Inconsistency of Loop-Specific Approach for Δ HbA1c (%)

| Loop | IF | seIF | z_value | p_value | CI_95 |
| --- | --- | --- | --- | --- | --- |
| A-H-J | 0.36 | 0.038 | 9.56 | 0 | (0.29,0.43) |
| A-F-K | 0.334 | 0.307 | 1.088 | 0.276 | (0.00,0.94) |
| E-F-H | 0.291 | 0.037 | 7.96 | 0 | (0.22,0.36) |
| A-D-K | 0.251 | 0.311 | 0.807 | 0.42 | (0.00,0.86) |
| A-F-H | 0.228 | 0.31 | 0.735 | 0.462 | (0.00,0.84) |
| A-I-K | 0.205 | 0.329 | 0.623 | 0.533 | (0.00,0.85) |
| F-I-K | 0.13 | 0.01 | 12.96 | 0 | (0.11,0.15) |
| A-I-J | 0.115 | 0.143 | 0.808 | 0.419 | (0.00,0.40) |
| D-I-K | 0.07 | 0.099 | 0.708 | 0.479 | (0.00,0.26) |
| A-D-I | 0.06 | 0.182 | 0.332 | 0.74 | (0.00,0.42) |
| F-H-I-J | 0.05 | 0.009 | 5.857 | 0 | (0.03,0.07) |
| A-G-I | 0.05 | 0.272 | 0.183 | 0.855 | (0.00,0.58) |
| A-E-F | 0.046 | 0.281 | 0.162 | 0.871 | (0.00,0.60) |
| A-E-H | 0.042 | 0.041 | 1.028 | 0.304 | (0.00,0.12) |
| A-F-I | 0.014 | 0.252 | 0.057 | 0.954 | (0.00,0.51) |

Note: A: Placebo; D: Lixi20μg; E: Tirze15mg; F: Dula1.5mg; G: Oralsema14mg; H: Sema1.0mg; I: Lira1.8mg; J: Weekly-Exe2.0mg; K: Daily-Exe10μg. IF p-value＞0.05 or CI_95 include 0 was defined as not significant inconsistent.

**4.2.2 The Inconsistency of Loop-Specific Approach for Δ Weight (kg)**

| Loop | IF | seIF | z_value | p_value | CI_95 |
| --- | --- | --- | --- | --- | --- |
| A-E-H | 2.085 | 0.293 | 7.126 | 0 | (1.51,2.66) |
| A-H-J | 1.85 | 0.257 | 7.191 | 0 | (1.35,2.35) |
| A-F-H | 1.418 | 0.935 | 1.518 | 0.129 | (0.00,3.25) |
| E-F-H | 1.125 | 0.19 | 5.925 | 0 | (0.75,1.50) |
| A-D-I | 0.895 | 0.952 | 0.94 | 0.347 | (0.00,2.76) |
| D-I-K | 0.84 | 0.462 | 1.818 | 0.069 | (0.00,1.75) |
| A-G-I | 0.667 | 1.337 | 0.499 | 0.618 | (0.00,3.29) |
| A-E-F | 0.627 | 0.819 | 0.766 | 0.444 | (0.00,2.23) |
| A-F-I | 0.601 | 0.826 | 0.728 | 0.467 | (0.00,2.22) |
| F-I-K | 0.57 | 0.043 | 13.106 | 0 | (0.48,0.66) |
| A-D-K | 0.302 | 1.229 | 0.246 | 0.806 | (0.00,2.71) |
| A-I-K | 0.172 | 1.185 | 0.145 | 0.885 | (0.00,2.49) |
| A-F-K | 0.106 | 1.236 | 0.086 | 0.932 | (0.00,2.53) |
| A-I-J | 0.099 | 1.111 | 0.089 | 0.929 | (0.00,2.28) |
| F-H-I-J | 0.05 | 0.038 | 1.331 | 0.183 | (0.00,0.12) |

Note: A: Placebo; D: Lixi20μg; E: Tirze15mg; F: Dula1.5mg; G: Oralsema14mg; H: Sema1.0mg; I: Lira1.8mg; J: Weekly-Exe2.0mg; K: Daily-Exe10μg. IF p-value＞0.05 or CI_95 include 0 was defined as not significant inconsistent.

**4.2.3 The Inconsistency of Loop-Specific Approach for the Rate of Adverse Events**

| Loop | IF | seIF | z_value | p_value | CI_95 |
| --- | --- | --- | --- | --- | --- |
| A-H-J | 2.201 | 1.162 | 1.894 | 0.058 | (0.00,4.48) |
| A-F-H | 1.848 | 1.175 | 1.573 | 0.116 | (0.00,4.15) |
| A-E-H | 1.25 | 1.172 | 1.066 | 0.286 | (0.00,3.55) |
| A-F-K | 0.797 | 0.509 | 1.565 | 0.118 | (0.00,1.79) |
| F-I-K | 0.559 | 0.339 | 1.647 | 0.1 | (0.00,1.22) |
| H-I-J | 0.54 | 0.328 | 1.647 | 0.099 | (0.00,1.18) |
| D-I-K | 0.466 | 0.354 | 1.319 | 0.187 | (0.00,1.16) |
| A-G-I | 0.449 | 0.349 | 1.287 | 0.198 | (0.00,1.13) |
| A-E-F | 0.292 | 0.68 | 0.43 | 0.667 | (0.00,1.63) |
| E-F-H | 0.257 | 0.544 | 0.472 | 0.637 | (0.00,1.32) |
| A-I-K | 0.244 | 0.813 | 0.3 | 0.764 | (0.00,1.84) |
| A-F-I | 0.164 | 0.533 | 0.308 | 0.758 | (0.00,1.21) |
| A-D-I | 0.135 | 0.489 | 0.276 | 0.783 | (0.00,1.09) |
| A-D-K | 0.045 | 0.674 | 0.067 | 0.946 | (0.00,1.37) |
| A-I-J | 0.021 | 0.729 | 0.029 | 0.977 | (0.00,1.45) |

Note: A: Placebo; D: Lixi20μg; E: Tirze15mg; F: Dula1.5mg; G: Oralsema14mg; H: Sema1.0mg; I: Lira1.8mg; J: Weekly-Exe2.0mg; K: Daily-Exe10μg. IF p-value＞0.05 or CI_95 include 0 was defined as not significant inconsistent.

**4.2.4 The Inconsistency of Loop-Specific Approach for the Rate of Serious Adverse Events**

| Loop | IF | seIF | z_value | p_value | CI_95 |
| --- | --- | --- | --- | --- | --- |
| A-H-J | 2.451 | 1.804 | 1.358 | 0.174 | (0.00,5.99) |
| F-H-I-J | 1.965 | 0.832 | 2.363 | 0.018 | (0.33,3.59) |
| A-F-H | 1.584 | 1.636 | 0.968 | 0.333 | (0.00,4.79) |
| A-D-I | 1.272 | 0.665 | 1.914 | 0.056 | (0.00,2.57) |
| A-E-H | 1.167 | 1.844 | 0.633 | 0.527 | (0.00,4.78) |
| E-F-H | 1.128 | 1.04 | 1.084 | 0.278 | (0.00,3.17) |
| A-I-K | 0.787 | 0.603 | 1.304 | 0.192 | (0.00,1.97) |
| A-F-I | 0.697 | 0.681 | 1.025 | 0.306 | (0.00,2.03) |
| A-I-J | 0.4 | 0.997 | 0.401 | 0.688 | (0.00,2.35) |
| A-D-K | 0.38 | 0.691 | 0.55 | 0.582 | (0.00,1.73) |
| F-I-K | 0.151 | 0.845 | 0.179 | 0.858 | (0.00,1.81) |
| D-I-K | 0.105 | 0.869 | 0.121 | 0.904 | (0.00,1.81) |
| A-E-F | 0.072 | 1.369 | 0.053 | 0.958 | (0.00,2.75) |
| A-G-I | 0.069 | 0.521 | 0.133 | 0.894 | (0.00,1.09) |
| A-F-K | 0.037 | 0.607 | 0.06 | 0.952 | (0.00,1.23) |

Note: A: Placebo; D: Lixi20μg; E: Tirze15mg; F: Dula1.5mg; G: Oralsema14mg; H: Sema1.0mg; I: Lira1.8mg; J: Weekly-Exe2.0mg; K: Daily-Exe10μg. IF p-value＞0.05 or CI_95 include 0 was defined as not significant inconsistent.

**4.2.5 The Inconsistency of Loop-Specific Approach for the Rate of Hypoglycemic Events**

| Loop | IF | seIF | z_value | p_value | CI_95 |
| --- | --- | --- | --- | --- | --- |
| A-D-K | 1.909 | 1.466 | 1.302 | 0.193 | (0.00,4.78) |
| A-H-J | 1.079 | 1.888 | 0.571 | 0.568 | (0.00,4.78) |
| A-D-I | 0.97 | 1.102 | 0.88 | 0.379 | (0.00,3.13) |
| D-F-I-K | 0.771 | 0.941 | 0.82 | 0.412 | (0.00,2.62) |
| A-E-H | 0.696 | 1.921 | 0.362 | 0.717 | (0.00,4.46) |
| E-F-H | 0.438 | 1.258 | 0.348 | 0.728 | (0.00,2.90) |
| A-F-I | 0.421 | 0.638 | 0.659 | 0.51 | (0.00,1.67) |
| A-E-F | 0.308 | 1.448 | 0.213 | 0.831 | (0.00,3.15) |
| A-F-H | 0.294 | 1.821 | 0.161 | 0.872 | (0.00,3.86) |
| A-G-I | 0.087 | 1.285 | 0.068 | 0.946 | (0.00,2.61) |
| A-F-K | 0.041 | 0.966 | 0.042 | 0.966 | (0.00,1.93) |

Note: A: Placebo; D: Lixi20μg; E: Tirze15mg; F: Dula1.5mg; G: Oralsema14mg; H: Sema1.0mg; I: Lira1.8mg; J: Weekly-Exe2.0mg; K: Daily-Exe10μg. IF p-value＞0.05 or CI_95 include 0 was defined as not significant inconsistent.

**4.2.6 The Inconsistency of Loop-Specific Approach for the Rate of AE Withdraw**

| Loop | IF | seIF | z_value | p_value | CI_95 |
| --- | --- | --- | --- | --- | --- |
| A-E-H | 3.114 | 1.704 | 1.827 | 0.068 | (0.00,6.45) |
| A-F-H | 2.583 | 1.618 | 1.597 | 0.11 | (0.00,5.75) |
| A-H-J | 1.679 | 1.762 | 0.953 | 0.341 | (0.00,5.13) |
| A-I-J | 1.391 | 1.629 | 0.854 | 0.393 | (0.00,4.58) |
| A-E-F | 1.048 | 1.883 | 0.557 | 0.578 | (0.00,4.74) |
| A-D-K | 0.593 | 0.641 | 0.925 | 0.355 | (0.00,1.85) |
| E-F-H | 0.564 | 0.679 | 0.831 | 0.406 | (0.00,1.90) |
| A-F-K | 0.464 | 0.873 | 0.532 | 0.595 | (0.00,2.17) |
| A-F-I | 0.461 | 1.072 | 0.43 | 0.667 | (0.00,2.56) |
| A-G-I | 0.458 | 0.755 | 0.606 | 0.544 | (0.00,1.94) |
| A-I-K | 0.288 | 0.98 | 0.294 | 0.769 | (0.00,2.21) |
| A-D-I | 0.218 | 1.032 | 0.211 | 0.833 | (0.00,2.24) |
| F-I-K | 0.211 | 0.669 | 0.316 | 0.752 | (0.00,1.52) |
| D-I-K | 0.059 | 0.549 | 0.108 | 0.914 | (0.00,1.14) |
| F-H-I-J | 0.044 | 0.637 | 0.069 | 0.945 | (0.00,1.29) |

Note: A: Placebo; D: Lixi20μg; E: Tirze15mg; F: Dula1.5mg; G: Oralsema14mg; H: Sema1.0mg; I: Lira1.8mg; J: Weekly-Exe2.0mg; K: Daily-Exe10μg. IF p-value＞0.05 or CI_95 include 0 was defined as not significant inconsistent.
